# Response Letter regarding the Article “Comparison between Fixation with Smooth Kirschner Wire and Cannulated Screws in Displaced Fractures of the Lateral Humeral Condyle in Children”

**DOI:** 10.1055/s-0044-1779323

**Published:** 2024-01-31

**Authors:** Alberto Daniel Navarro Vergara, Alberto Navarro Fretes

**Affiliations:** 1 Hospital de Trauma "Manuel Giagni", Hospital Central de IPS, Asunción, Paraguay; 2 Departamento de Ortopedia Infantil do HC-IPS, Asunción, Paraguay

I sincerely appreciate the opportunity to respond to the constructive criticism that has been raised regarding our work entitled " Comparison between Fixation with Smooth Kirschner Wire and Cannulated Screws in Displaced Fractures of the Lateral Humeral Condyle in Children", and would like to express my appreciation for the time and attention dedicated to reviewing our work. We have taken into consideration each of the points raised and, after a thorough analysis, we wish to provide a detailed response to each of the observations:

1. Exclusive use of the Weiss classification: We welcome the suggestion to consider integrating other classifications, such as Milch and Jacob, to provide a more complete perspective on lateral humeral condyle fractures. We agree that a combination of classifications could enrich our work and allow for a better understanding of the anatomy, displacement and outcomes of these fractures. 1 2 In future research, we will strive to incorporate multiple classifications for a more complete analysis.
2. Failure to use internal oblique radiographs (IOR): We appreciate your recommendation to use internal oblique radiographs to measure displacement of lateral condyle fractures. We understand that this view could provide additional information 3 and a more accurate assessment of displacement, 4 and we are committed to including it in future studies to improve the reliability of our results.
3. Choice of treatment for type II Weiss fractures: We recognize the relevance of your question about why we chose to perform open reduction in certain cases of type II Weiss fractures that, according to previous studies, could have been treated with closed reduction and percutaneous pinning (CRPP). Unfortunately, in these cases, there were specific factors that required a more invasive surgical approach, such as the time of evolution and failure to achieve reduction after attempting preoperative manipulation. 5 However, we understand the importance of always considering less invasive treatment options and will certainly justify our decisions in future publications.
4. Assessment of articular cartilage integrity: We appreciate the reference to studies that used arthrograms or ultrasound to assess the integrity of articular cartilage in lateral condyle fractures. 1 6 We recognize the value of this information in clinical decision making and future studies, and will strive to include these assessments to determine the most appropriate treatment options based on the integrity of the articular cartilage.
5. Inconsistencies in the study: We appreciate you pointing out these inconsistencies in our work. We recognize that clarity and transparency are fundamental in any investigation and we apologize for discrepancies in the description of our methodology. In future publications, we will ensure that we provide a clear and consistent description of our study design and treatment-related decision making.
6. Assessment of functional outcome and complications: We took into consideration your suggestion to include more detailed data on observed range of motion and the time required for function to fully recover. 7 8 We understand the importance of assessing long-term functional outcome timing and incidence of complications and we will strive to provide more complete information in future publications.

In summary, we sincerely welcome your criticisms and suggestions, as they allow us to improve the quality and relevance of our research. We took note of each point and are committed to addressing them in future research to strengthen the knowledge base in the field of lateral humeral condyle fractures. We thank you again for your time and consideration, and we hope that our future publications will be of greater value to the scientific community and readers of Revista Brasileira de Ortopedia.
