# Supplementary material for: Response Letter regarding the Article “Comparison between Fixation with Smooth Kirschner Wire and Cannulated Screws in Displaced Fractures of the Lateral Humeral Condyle in Children”
Source: Rev Bras Ortop (Sao Paulo). 2024 Jan 31;59(6):e1010–1. doi: 10.1055/s-0044-1779323 (PMC11663049; doi:10.1055/s-0044-1779323)
Supplement: Related texts

# Research article: Carta resposta referente ao artigo “Comparison between Fixation with Smooth Kirschner Wire and Cannulated Screws in Displaced Fractures of the Lateral Humeral Condyle in Children”

Agradeço sinceramente a oportunidade de responder às críticas construtivas que foram levantadas em relação ao nosso trabalho intitulado "Comparison between Fixation with Smooth Kirschner Wire and Cannulated Screws in Displaced Fractures of the Lateral Humeral Condyle in Children", e gostaria de expressar meu agradecimento pelo tempo e atenção dedicados à revisão de nosso trabalho. Levamos em consideração cada um dos pontos levantados e, após uma análise minuciosa, desejamos fornecer uma resposta detalhada a cada uma das observações:

1. Uso exclusivo da classificação de Weiss: Acolhemos a sugestão de considerar a integração de outras classificações, como Milch e Jacob, para fornecer uma perspectiva mais completa sobre as fraturas laterais do côndilo do úmero. Concordamos que uma combinação de classificações poderia enriquecer nosso trabalho e permitir um melhor entendimento da anatomia, desvio e resultados dessas fraturas. 1 2 Em pesquisas futuras, nos esforçaremos para incorporar múltiplas classificações para uma análise mais completa.
2. Ausência de uso de radiografias oblíquas internas (IOR): Agradecemos sua recomendação de usar radiografias oblíquas internas para medir o deslocamento das fraturas do côndilo lateral. Entendemos que essa visão poderia fornecer informações adicionais 3 e uma avaliação mais precisa do deslocamento, 4 e estamos comprometidos em incluí-la em estudos futuros para melhorar a confiabilidade de nossos resultados.
3. Escolha do tratamento para as fraturas de Weiss tipo II: Reconhecemos a relevância de sua pergunta sobre por que optamos por realizar a redução aberta em certos casos de fraturas de Weiss tipo II que, de acordo com estudos anteriores, poderiam ter sido tratadas com redução fechada e fixação percutânea (PRCR). Infelizmente, nesses casos, houve fatores específicos que necessitaram de uma abordagem cirúrgica mais invasiva, como o tempo de evolução e a não obtenção da redução após a tentativa de manipulação pré-operatória. 5 No entanto, entendemos a importância de sempre considerar as opções de tratamento menos invasivas e certamente justificaremos nossas decisões em futuras publicações.
4. Avaliação da integridade da cartilagem articular: Agradecemos a referência a estudos que utilizaram artrogramas ou ultrassom para avaliar a integridade da cartilagem articular em fraturas do côndilo lateral. 1 6 Reconhecemos o valor dessas informações na tomada de decisão clínica e em estudos futuros, e nos esforçaremos para incluir essas avaliações para determinar as opções de tratamento mais apropriadas com base na integridade da cartilagem articular.
5. Inconsistências no estudo: Agradecemos que você aponte essas inconsistências em nosso trabalho. Reconhecemos que clareza e transparência são fundamentais em qualquer investigação e pedimos desculpas por discrepâncias na descrição de nossa metodologia. Em publicações futuras, garantiremos que fornecemos uma descrição clara e consistente do desenho do nosso estudo e da tomada de decisões relacionadas ao tratamento.
6. Avaliação do resultado funcional e complicações: Levamos em consideração sua sugestão de incluir dados mais detalhados sobre a amplitude de movimento observada e o tempo necessário para que a função se recupere totalmente. 7 8 Entendemos a importância de avaliar o resultado funcional a longo prazo e a incidência de complicações e nos esforçaremos para fornecer informações mais completas em publicações futuras.

Em resumo, acolhemos sinceramente suas críticas e sugestões, pois elas nos permitem melhorar a qualidade e a relevância de nossa pesquisa. Tomamos nota de cada ponto e estamos comprometidos em abordá-los em pesquisas futuras para fortalecer a base de conhecimento no campo das fraturas laterais do côndilo do úmero. Agradecemos novamente por seu tempo e consideração, e esperamos que nossas futuras publicações sejam de maior valor para a comunidade científica e leitores da Revista Brasileira de Ortopedia.
